# Neuroimaging in PRUNE1 syndrome: a mini-review of the literature

**DOI:** 10.3389/fneur.2023.1301147

**Published:** 2023-12-21

**Authors:** Giovanna Scorrano, Laura Battaglia, Rossana Spiaggia, Antonio Basile, Stefano Palmucci, Pietro Valerio Foti, Emanuele David, Franco Marinangeli, Ilaria Mascilini, Antonio Corsello, Francesco Comisi, Alessandro Vittori, Vincenzo Salpietro

**Affiliations:** ^1^Department of Biotechnological and Applied Clinical Sciences, University of L'Aquila, L'Aquila, Italy; ^2^Department of Medical Surgical Sciences and Advanced Technologies "GF Ingrassia", University Hospital Policlinic "G. Rodolico-San Marco", Catania, Italy; ^3^Department of Anesthesia, Critical Care and Pain Therapy, University of L'Aquila, L'Aquila, Italy; ^4^Department of Anesthesia and Critical Care, ARCO ROMA, Ospedale Pediatrico Bambino Gesù IRCCS, Rome, Italy; ^5^Department of Pediatrics, University of Milan, Milan, Italy; ^6^Pediatrics Department, Ospedale Microcitemico, Cagliari, Italy; ^7^Department of Neuromuscular Disorders, UCL Queen Square Institute of Neurology, London, United Kingdom

**Keywords:** *PRUNE1*, neurodevelopmental disorder, enzymatic activity, neurogenesis, delayed myelination

## Abstract

Prune exopolyphosphatase 1 (PRUNE1) is a short-chain phosphatase that is part of the aspartic acid-histidine-histidine (DHH) family of proteins. PRUNE1 is highly expressed in the central nervous system and is crucially involved in neurodevelopment, cytoskeletal rearrangement, cell migration, and proliferation. Recently, biallelic *PRUNE1* variants have been identified in patients with neurodevelopmental disorders, hypotonia, microcephaly, variable cerebral anomalies, and other features. *PRUNE1* hypomorphic mutations mainly affect the DHH1 domain, leading to an impactful decrease in enzymatic activity with a loss-of-function mechanism. In this review, we explored both the clinical and radiological spectrum related to *PRUNE1* pathogenic variants described to date. Specifically, we focused on neuroradiological findings that, together with clinical phenotypes and genetic data, allow us to best characterize affected children with diagnostic and potential prognostic implications.

## 1 Introduction

Prune exopolyphosphatase 1 (PRUNE1) is a component of the aspartic acid-histidine-histidine (DHH) family of proteins with hydrolysis activity on short-chain polyphosphates (1–3). The DHH catalytic domain is located at the N-terminal end of the protein and consists of highly conserved loading residues that bind metal cofactors during interaction with substrates (1, 4). PRUNE1 is highly expressed in the central nervous system during fetal development and plays a crucial role in cell migration and proliferation, presumably through interaction at the C-terminal end with proteins involved in cytoskeletal rearrangement (3, 5–8). Probably implicated in the hydrolysis of the second messenger cyclic adenosine monophosphate (cAMP), PRUNE1 also modulates many sub-cellular processes and appears to be critical for embryogenesis in mouse models (1, 3, 9). Interestingly, both *PRUNE1* −/− mice and *PRUNE1* conditional knockout mice generated by cell-specific inactivation of *PRUNE1* died within embryonic day 12 and exhibited cardiac anomalies, vascular remodeling, and hematopoietic defects, in addition to anomalies in the differentiation of trophoblasts (1, 3). These mice also presented with Purkinje cell degeneration during embryonic age, with the loss of these same cells (>90%) within 2 months of age (3).

Moreover, PRUNE1 has been involved in postnatal neurodevelopment and metastatic processes, being implicated in cell cycle and motility (1, 3, 8–10). Specifically, mutations in *PRUNE1* have been related to different metastatic subgroups of medulloblastoma. It was observed that the PRUNE1 protein, which is highly expressed in metastatic medulloblastoma, stimulates the TGF-β pathway with consequent upregulation of OTX2 and SNAIL and PTEN inhibition. In this context, two different substrates (a small pyrimido-pyrimidine derivative, AA7.1, and a competitive permeable peptide) inhibited the PRUNE1/TGF-β/OTX2/PTEN axis and prevented tumor growth and metastatic dissemination in a functional model (5, 11). The overexpression of PRUNE1 has also been involved in the oncogenesis and metastatic progression of thyroid cancer, esophageal squamous cell carcinoma, gastric cancer, colorectal cancer, non-small cell lung cancer, breast cancer and triple-negative breast cancer, hepatocellular carcinoma, and neuroblastoma. In particular, silencing PRUNE1 in human cancer cells impaired cell motility and metastasis formation (5).

Autosomal recessive pathogenic variants of *PRUNE1* have been associated with microcephaly, hypotonia, and variable brain anomalies (NMIHBA, MIM #617481) (1, 12). In recent years, the clinical-radiological spectrum related to PRUNE1 has expanded to include craniofacial anomalies, skeletal muscle and articular impairment, neuropathy, profound global developmental delay, cortical and cerebellar atrophy, white matter disease, corpus callosum abnormalities, and seizures (3, 13–16).

The aim of this review is to delineate the emerging clinical-radiological spectrum related to *PRUNE1* pathogenic variants, focusing on brain magnetic resonance imaging signs, better characterize the disorder, and provide clinicians with a helpful instrument for diagnostic work-up.

## 2 Genetic features

The *PRUNE1* gene is situated on chromosome 1q21.3 and encodes a protein of 453 amino acids. The PRUNE1 protein is involved in synaptogenesis, neurite growth, and neurogenesis and includes two domains, DHH (20–172 amino acids) and DHHA2 (215–359 amino acids) (6, 17).

*PRUNE1* pathogenic variants present pan-ethnic expression and are related to an expanding neuroradiological phenotype. Those described to date are missense, start-loss, nonsense, deletions, loss-of-function splicing, homozygous, and compound heterozygous, inherited in an autosomal recessive pattern. Interestingly, the expression of homozygous *PRUNE1* variants was more pronounced in those populations with high rates of consanguineous marriages, and the majority of homozygous patients carried a missense variant, suggesting that *PRUNE1*-related disorders are mainly associated with hypomorphic alleles (1, 13). Moreover, rare severe alleles from recent ancestors were associated with a relevant effect on PRUNE syndrome, greater than common variants reported in populations. If these rare alleles congregate in homozygosis, a founder effect and/or a high consanguinity rate may play a crucial role (1, 18).

To date, 64 patients with *PRUNE1*-related disorders have been described, with no evidence of a clear genotype–phenotype correlation. Indeed, patients harboring the same variant allele displayed a variable severity of clinical phenotype (1).

**Figure 1:**
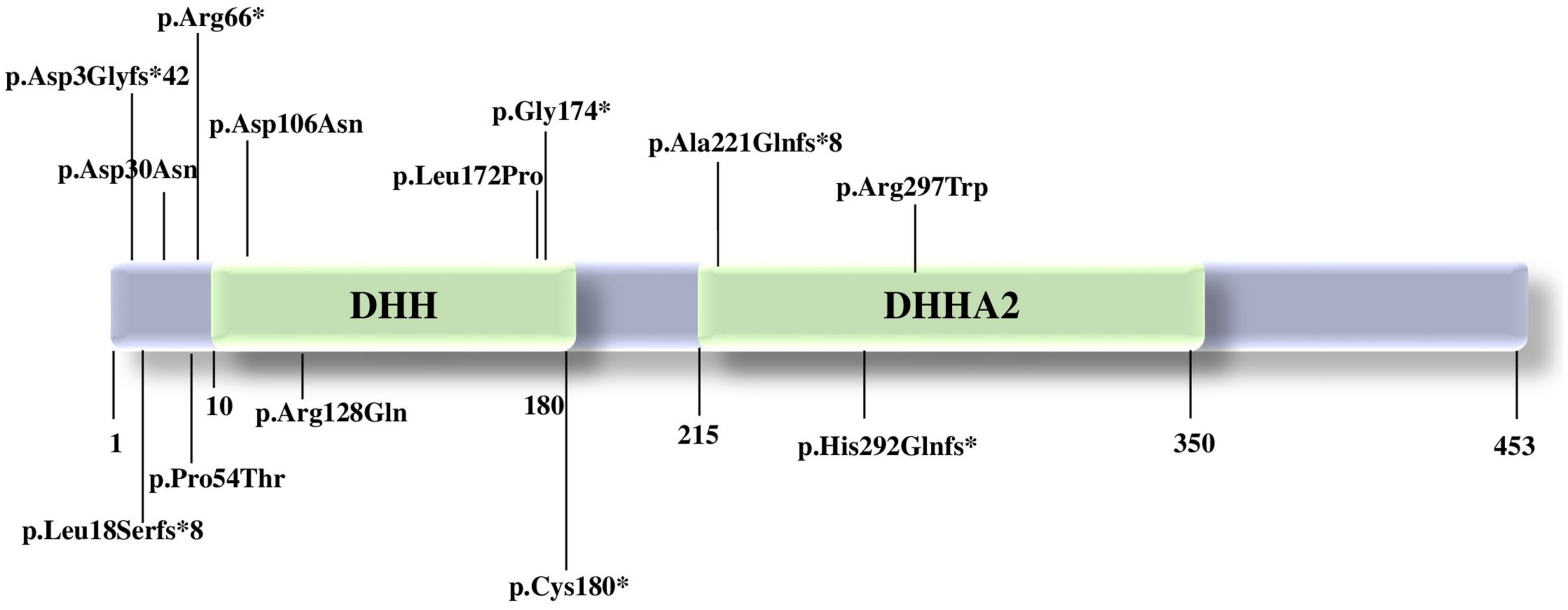
Pathogenic variants in *PRUNE1* reported to date. PRUNE1 figure protein with the catalytic domains and the pathogenic variants described so far.

The majority of the pathogenic variants reported seems to affect the DHH domain, leading to a variable loss of protein function (17) (Figure 1). The DHH disruption impairs the protein structure and the interaction with divalent metal ions, preventing substrate binding (1). Specifically, according to a synergistic mechanism, the metal ion binding in the active site of the protein accelerates the substrate binding to PRUNE1, which in turn increases the enzymatic affinity for the metal ion. Indeed, comparative modeling of variants affecting one of the three highly conserved amino acids of the DHH domain (Asp-His-His) showed a relevant decrease in enzymatic activity, with subsequent variable loss of function of the PRUNE1 protein (1, 10, 17).

Homozygous variants p.(Met1?) (17), p.Asp106Asn (12), c.521-2A>G: IVS4-2A>G (19), p.D30N, p.D106N, and compound heterozygous variants p.R128Q and p.G174X (12) affecting the catalytic DHH domain showed neurological features such as developmental delay, intellectual disability with speech disorder, cerebral and cerebellar atrophy, hypotonia, spastic quadriplegia, microcephaly, and seizures, with decreased enzymatic and short-chain exopolyphosphate activity.

Concurrently, patients carrying the homozygous variant c.521-2A>G: IVS4-2A>G, p.(Met1?), p.Cys180* presented a neurological phenotype without microcephaly. Both organoid studies and the expansion of the clinical spectrum will be able to clarify the impact of each variant and possibly better define a genotype–phenotype correlation (17).

PRUNE1 appears crucial in mouse embryogenesis, and *PRUNE1*-null mouse models showed cardiac hypoplasia and profound vascular defects, not reported in humans with *PRUNE1*-related disorders despite a high cardiac expression in human adults (1). Moreover, homozygous deletion of *PRUNE1* results in embryonic lethality in the mouse model but not in humans. Although the role of *PRUNE1* in embryonic development is currently not known, studies to date support a pivotal role for *PRUNE1* in human neuronal survival during development. Further functional studies will better elucidate the molecular mechanisms of *PRUNE1*-related disorders.

## 3 Clinical features

Considering the 64 patients with *PRUNE1*-related disorders described to date, no specific genotype–phenotype correlation has emerged. PRUNE1 is highly expressed in the cortex, hippocampus, midbrain, and cerebellum of the developing brain. Affected patients are frequently born after an uncomplicated pregnancy and present with an unremarkable perinatal period. However, during the first months of life, they usually manifest severe global neurodevelopmental delays and comorbidities that worsen over time. Specifically, they do not achieve neurodevelopmental milestones (~90%) and exhibit moderate to profound intellectual disability (83%), with speech delay (~78%), hypotonia (42%), spastic quadriplegia (22%), microcephaly (61%), and seizures (60%) (17). Language is often absent, with patients communicating with grunts and cries. Axial hypotonia with distal hypertonia/spasticity and brisk tendon reflexes characterize these patients, and peripheral neuropathy, reduced nerve conduction velocity, and/or spinal motor neuron involvement have also been described (1, 20). Patients often present with seizures starting at a mean age of 6 months. These include infantile spasms, focal and generalized gelastic, myoclonic, clonic, and/or tonic seizures refractory to antiseizure medications (1, 21–24). In this context, the electroencephalogram (EEG) documents variable discharges, progressive development of hypsarrythmia, and/or slowed background activity as the epileptic encephalopathy worsens. Furthermore, vision problems such as optic atrophy, esotropia, cortical blindness, bilateral rudimentary iris strands, congenital cataracts, saccadic eye movements, and nystagmus have been reported and are often present after birth (1, 8, 13, 19, 20, 23). Recently, gastrointestinal disorders such as dysphagia and gastrointestinal reflux have been described, usually followed by inadequate oral caloric intake and failure to thrive, requiring nasogastric tube feeding or gastrotomy tube placement (1, 13, 20). Skeletal issues such as kyphosis and scoliosis have also been observed (1, 20, 23). Dysmorphic features described include craniofacial anomalies such as a sloping or high forehead, large prominent or low-set ears, prominent eyes, a narrow, high-arched palate, epicanthus, hypertelorism, a flat nasal bridge, detached and hypoplastic nipples, hirsutism, abnormal dentition with widely spaced teeth, micro-retrognathia, plagiocephaly, bitemporal narrowing, brachycephaly, and brachydactyly. Other findings, then, include arthrogryposis/contractures, hypertrophic cardiomyopathy, pectus excavatum, clubfoot, bilateral talipes equinovarus, respiratory distress, exaggerated startle, bilateral Babinski signs, and sustained ankle clonus (1).

## 4 Imaging findings

Brain imaging plays a crucial role in the diagnostic workup of *PRUNE1*-related disorders. However, previous literature does not entirely elucidate the distinct MRI anomalies associated with PRUNE1.

**Figure 2:**
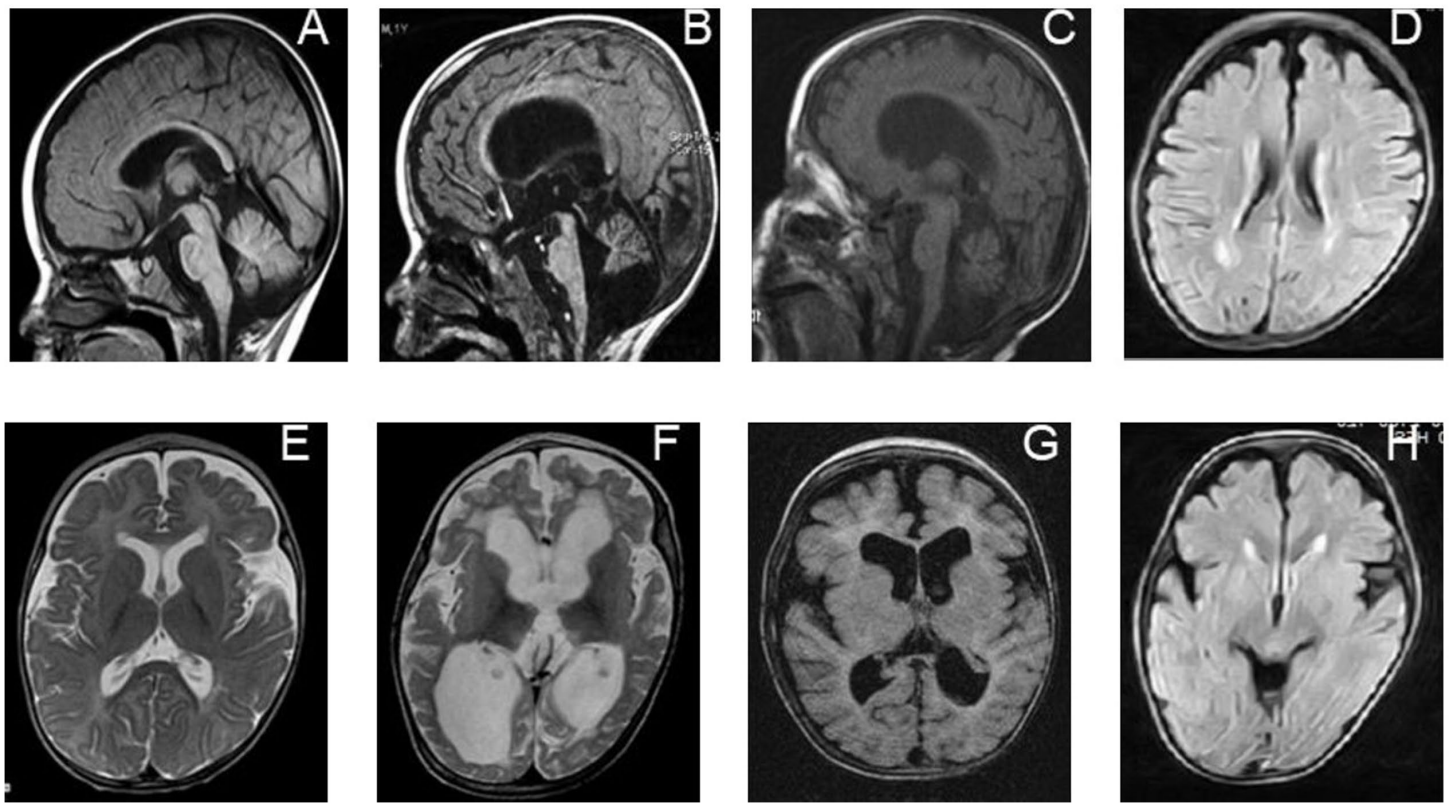
Neuroradiological features of individuals with biallelic *PRUNE1* mutations. Sagittal T1-weighted images of a *PRUNE1*-affected patient performed at 6 months of age **(A)** and 16 months of age **(B)** show progressive global brain atrophy, but more specifically evidence of cerebellar and brainstem atrophy, which is out of proportion to the cerebral atrophy. **(E,F)** Axial T2-weighted images performed at 6 months of age **(E)** and 16 months of age **(F)** in the same child, showing progressive diffuse white matter abnormalities along with progressive brain atrophy. **(C)** Axial inversion recovery and **(G)** sagittal T1-weighted MRI sequences performed in the patient at 24 months of age show generalized brain volume loss, but with specific evidence of cerebellar atrophy and a diffuse white matter signal abnormality as was seen in her sibling **(D,H)**.

However, specific MRI features related to *PRUNE1* pathogenic variants have emerged over the years, often including brain malformations (Figure 2; Table 1). Our aim is to analyze the *PRUNE1*-related MRI features described to date in order to establish a distinct radiological pattern.

**Table 1:** MRI Features of *PRUNE1* affected patients reported to date.

MRI Features	Karaca et al. (12)	Costain et al. (19)	Zollo et al. (8)	Karakaya et al. (23)	Iacomino et al. (22)	Alfadhel et al. (13)	Hartley et al. (14)	Hiroyuki et al. (21)	Koko et al. (25)	Gholizadeh et al. (17)
Cerebral atrophy	+	+	+	+	+	+	−	+	+	+
Cerebellar atrophy	+	+	+	+			−	+	+	+
Delayed myelination			+	+		+	−			+
Hypoplasia of the corpus callosum		+	+		+	+	−	+	+	+
White matter disease		+	+		+		−	+		
Microcephaly	+		+	+		+	−			
White matter hyperintensities (T2)		+					−	+	+	
Optic atrophy			+	+			−			
Progression of abnormalities with age			+		+		−			

In 2015, Karaca et al. performed a study including 208 patients from 128 mainly consanguineous families with congenital brain malformations and/or intellectual disabilities. In four families, potentially deleterious variants of the *PRUNE1* gene, associated with intellectual disability, brain malformations, and cortical dysplasia, were identified. Specifically, a homozygous missense variant (NM_021222: c.G316A, p.D106N) in the *PRUNE1* gene was detected in two apparently unrelated families from neighboring villages in eastern Turkey. Both individuals affected by this variant showed microcephaly, frontotemporal cortical atrophy, and cerebellar atrophy on MRI. Furthermore, a rare homozygous missense variant (NM_02122:c.G88A:p.D30N) was described in an 18-month-old male patient with cerebral and cerebellar atrophy, microcephaly, and severe developmental delay. In four unrelated families from the United States, compound heterozygous missense variants (NM_021222: c.G383A:p.R128Q and NM_021222: c.G520T:p.G174X) were detected and were associated with severe developmental delay, regression, seizures, microcephaly, and brain atrophy (12).

In 2016, Costain et al. presented the case of a 2-year-old boy with a complex neurological phenotype and abnormalities detected on brain MRI. Upon re-evaluation of clinical whole-exome sequencing data, a homozygous, likely pathogenic splicing variant in *PRUNE1* (c.521-2A>G) was identified. The patient was born to non-consanguineous parents of Cree (maternal) and Ojibwe-Cree (paternal) descent. After birth, he developed central hypoventilation, requiring assisted ventilation. At neurological examination, bilateral talipes equinovarus (clubfoot) and generalized hypotonia were observed. Throughout his first year of life, additional issues emerged, including severe global developmental delay without regression, cortical blindness, infantile spasms characterized by hypsarrhythmia, and an active focus in the right posterior temporal region on the EEG. The patient underwent three MRI brain scans at the ages of 8 days, 7 weeks, and 10 months of age, each of which was unremarkable. The most recent MRI showed cortical atrophy and a small cerebellum. Bilateral cerebral white matter loss was detected with a thin corpus callosum and patchy T2 hyperintensity in the frontal and parietal regions. Areas of diffusion restriction and T2 hyperintensity were observed in the globus pallidus and subthalamic nuclei, likely related to the use of vigabatrin. Furthermore, time-of-flight MR angiography and single-voxel MR spectroscopy in the left basal ganglia region showed normal results (19).

In 2017, Zollo et al. described *PRUNE1* mutations in 13 individuals, ranging in age from 3 months to 21 years, belonging to extended families from Oman and Iran, with two smaller families from India and Italy. All of these families were affected with overlapping severe global neurodevelopmental delays. Neuroimaging revealed specific findings in affected individuals, including focal white matter changes, delayed myelination, cortical atrophy, a thin or underdeveloped corpus callosum, and cerebellar atrophy. In one case, serial MRI scans were performed and showed a severe progression of the disease over time. At 6 months of age, delayed myelination was observed, while a follow-up MRI at 18 months of age revealed widespread abnormalities in the white matter and progressive brain atrophy affecting the cerebral cortex and especially the cerebellum. Both Italian children presented with optic atrophy at 2 years of age (8).

Recently, Karakaya et al. described the new case of an affected individual from a consanguineous Turkish family with congenital hypotonia, secondary microcephaly, global developmental delay, and respiratory failure. Cranial MRI scans conducted at 14 months of age revealed cerebral and cerebellar atrophy, delayed myelination, and hypoplasia of the inferior vermis. Electromyography was also performed and documented the neurogenic involvement. At 38 months of age, the patient exhibited generalized muscle weakness, no head control, hypotonia, spasticity, and sustained ankle clonus. He also developed secondary microcephaly, with a head circumference of 48.0 cm, brachycephaly, large ears, optic atrophy, severe kyphoscoliosis, and flexion contractures. Due to his condition, he required permanent mechanical ventilation and a nasogastric tube for feeding problems (23).

Iacomino et al. reported the case of a patient with a homozygous *PRUNE1* mutation and spinal motor neuronal involvement observed by electrophysiologic exam and muscle biopsy. This 9-month-old child from Italy was born at term to healthy, non-consanguineous parents. At birth, the patient displayed distal joint contractures and profound hypotonia. Respiratory distress required an early intubation. From the age of 6 months, the patient experienced epileptic spasms, and the EEG revealed a slowed background and multifocal epileptic abnormalities, prominent in the left occipital and right temporal brain regions. Brain MRI documented diffuse cortical atrophy and severe white matter loss, with signal changes in the periventricular white matter and pons. Subsequent whole-exome sequencing identified a missense homozygous variant (c.316G>A, p.D106N) in the *PRUNE1* gene (NM_021222). These findings provide further insights and highlight the extensive involvement of both brain and spinal motor neurons in this patient (22).

Alfadhel M. et al. described two affected girls, aged 12 and 30 months, from unrelated Saudi families. Both patients had the same missense mutation in *PRUNE1* (c.383G>A, p.Arg128Gln), which had not been reported before in the homozygous state. Patient 1 exhibited various dysmorphic features, and the neurological examination revealed central hypotonia and spastic quadriplegia with brisk tendon reflexes and clonus. MRI showed delayed myelination, a slightly abnormal shape of the corpus callosum, and mild frontal cerebral atrophy. Patient 2 had severe global developmental delay, axial hypotonia, appendicular spasticity, and microcephaly. A brain MRI showed a slightly abnormally shaped corpus callosum and slightly prominent CSF spaces anteriorly with normal myelination. Magnetic resonance spectroscopy was unremarkable (13).

Hartley J. et al. provided a description of the clinical and neuropathological features observed in nine Cree children from Manitoba, Canada. A homozygous *PRUNE1* mutation was identified as the underlying cause of the disease. Both the central and peripheral nervous systems were involved in these affected patients. The subjects exhibited hypotonia, contractures, and feeding difficulties. Notably, their MRI brain scans and head sizes were within the normal range, suggesting a neuromuscular origin for these manifestations (14).

In 2019, Hiroyuki Fujii et al. presented a unique case from Japan involving a reported *PRUNE1* mutation. A brain MRI revealed distinct imaging findings not previously reported. The patient was a 12-month-old girl, the first child of non-consanguineous Japanese parents. She exhibited global developmental delay, intellectual disability, hypotonia, spastic quadriparesis, and hyperreflexia. Brain MRIs displayed cerebral and cerebellar atrophy, a thin corpus callosum, white matter changes, and abnormal signal intensity in the brainstem. Additionally, she presented a transient lesion in the subcortical white matter of the brain, atrophy of the midbrain and pontine tegmentum, and abnormal signal intensity in the swollen putamen and medial portions of the thalamus, which emerged after the age of 4 years. At the same age, the patient underwent whole-exome sequencing (WES) analysis, which identified biallelic PRUNE1 variants. Specifically, compound missense heterozygous mutations were detected (c.[316G>A];[540T>A], p.[Asp106Asn];[Cys180*]) (21).

In 2021, Koko M. et al. reported five patients from two unrelated consanguineous Sudanese families who exhibited global neurodevelopmental delay, pyramidal symptoms with prominent flexion contractures, and extrapyramidal signs and symptoms (severe dystonia and bradykinesia). A homozygous splice variant (NM_021222.3:c.132 + 2 T > C), possibly related to an in-frame deletion in the DHH domain or premature truncation of the protein, was detected in these patients. Mild cortical, subcortical, and cerebellar atrophy, and a thin corpus callosum, were described on brain MRI in these affected patients. Additionally, periventricular subcortical white matter hyperintensities were detected in one individual (25).

Finally, in 2022, Gholizadeh MA et al. examined four individuals (two affected and two healthy) from a consanguineous Iranian family. Whole-exome sequencing revealed a start-loss pathogenic variant, NM_021222.3:c.3G>A; p.(Met1?), in the *PRUNE1* gene in two patients from this family. These patients presented with spastic quadriplegic cerebral palsy, hypotonia, developmental delay, intellectual disability, optic atrophy, and cerebellar atrophy. Brain MRI documented cortical atrophy, a thin corpus callosum, cerebellar hypoplasia, and delayed myelination (17).

## 5 Discussion

Genetic neurodevelopmental disorders (NDDs) include a variety of monogenic conditions with increasing clinical and genetic heterogeneity, characterized by various impairments in language, motor abilities, cognitive and behavioral development, and neurological comorbidities such as epilepsy and movement disorders (26–30). Over the years, studies based on NGS and omics-related sciences have revealed an expanding molecular complexity underlying NDDs (31–36). Many novel molecular factors have been identified with consequent advantages in terms of better definition of clinical phenotypes, valuable prognostic information, detailed imaging studies, and targeted therapies for the children affected by these conditions (37–42).

*PRUNE1* pathogenic variants have been associated with a widening clinical-radiological spectrum. To date, no clear genotype–phenotype correlation has been found, but functional and computational studies showed a loss-of-function pathogenic mechanism of *PRUNE1*. Indeed, nonsense, missense, deletion, start-loss, splicing, and truncating variants mainly affect the DHH domain with variable impairment and a decrease in protein enzymatic activity.

PRUNE1 is a multifunctional protein that mainly acts by hydrolyzing short-chain polyphosphates through metal ion interaction at the catalytic domain level, modulating several cellular processes such as cytoskeletal reorganization, migration, differentiation, neurogenesis, and synaptogenesis. Its complex regulation, along with its multiple interactions, largely explain the emerging clinical spectrum of *PRUNE1*.

Over the years, several studies have investigated the molecular mechanisms associated with *PRUNE1* overexpression in several metastatic cancers. Interestingly, it was noted that PRUNE1 regulates cell migration, motility, and adhesion through interaction with glycogen synthase kinase 3 (GSK-3)-binding protein, paxillin, vinculin, and phosphorylation of different substrates such as tyrosine phosphorylation of focal adhesion kinase (FAK) and H2-domain- and SH3 domain-containing proteins (p130Cas and Crk) (6). Specifically, GSK-3 and *PRUNE1* cooperate to disassemble focal adhesions and promote cell migration. Additionally, it has cyclic nucleotide phosphodiesterase activity and negatively regulates nm23-H1, a protein with antimetastatic activity. It has been observed that mutations in *PRUNE1* lead to a gain of phosphodiesterase activity, promoting metastasis, cancer aggressiveness, and proliferation. Moreover, *PRUNE1* upregulates several genes involved in metastatic processes and modulates the TGF-β/OTX2/PTEN axis with the same consequences as previously described (6). Additionally, it stimulates β-catenin and the secretion of Wnt3a, vimentin, and cytokines such as IL-17F. These results suggest that this protein could be considered as a potential marker of cancer aggressiveness and a target for future personalized treatments.

Polyphosphate seems to play a crucial role in cell metabolism in different species and has recently been described as neuroprotective against amyloid accumulation (43). Contextually, several mechanisms have been described to prevent amyloid toxicity. Presumably, polyphosphate directly controls amyloid formation and degradation. Concurrently, it may inhibit the uptake and diffusion of amyloid fibrils or increase their turnover. Additionally, low polyphosphate levels may be related to mitochondrial dysfunction, which is frequently associated with amyloidosis. Interestingly, polyphosphate levels decrease with age and appear to be low in patients with Alzheimer’s disease (44). Furthermore, polyphosphate has been observed to act as a gliotransmitter, leading to the strong activation of astrocytes. It has presumably played a pivotal role in modulating their signal transmission (45). Interestingly, *in vitro* studies have shown that patients with amyotrophic lateral sclerosis (ALS) have an overexpression of polyphosphate in astrocytes. Concurrently, motor neuron death was prevented by polyphosphate degradation, suggesting that this molecule could be considered as an ALS biomarker and therapeutic target (46).

Furthermore, PRUNE1 acts as a microtubule-associated protein (MAP) and promotes microtubule polymerization during mitosis and migration processes. Consistently, mutations in NMIHBA patients lead to delayed polymerization, disrupting proliferation and migration in the developing brain (5).

The variable loss of phosphatase activity in affected patients caused by pathogenic *PRUNE1* variants may shed light on potential targeted treatments, such as the use of molecules able to restore its enzymatic activities.

MRI radiological findings show that the majority of the subjects examined have delayed myelination, thin corpus callosum, and white matter abnormalities; in particular, brain magnetic resonance imaging showed an abnormal shape of the corpus callosum with thinning in the T1-weighted image, while in the T2-weighted image it showed mild frontal cerebral atrophy, prominent cerebellar atrophy, and delayed myelination. Imaging data represent a valuable instrument that, together with specific clinical features and genetic analysis, can best define *PRUNE1*-related disorders with diagnostic and potential prognostic implications.

We suggest that clinicians should consider the *PRUNE1* gene in the diagnostic workup of any child presenting with dysmorphic features, developmental delay, microcephaly, central hypotonia, peripheral spasticity, delayed myelination, cerebral atrophy, and thin corpus callosum.

## Author contributions

GS: Conceptualization, Data curation, Formal analysis, Funding acquisition, Investigation, Methodology, Project administration, Resources, Software, Supervision, Validation, Visualization, Writing – original draft, Writing – review & editing. LB: Conceptualization, Validation, Visualization, Writing – original draft. RS: Data curation, Methodology, Software, Writing – original draft. AB: Formal analysis, Investigation, Software, Writing – original draft. SP: Conceptualization, Data curation, Methodology, Writing – original draft. PF: Methodology, Software, Writing – original draft. ED: Conceptualization, Investigation, Methodology, Writing – original draft. FM: Conceptualization, Data curation, Software, Writing – original draft. IM: Conceptualization, Formal analysis, Methodology, Writing – original draft. AC: Conceptualization, Data curation, Software, Writing – original draft. FC: Data curation, Investigation, Methodology, Writing – original draft. AV: Writing – original draft, Writing – review & editing. VS: Conceptualization, Data curation, Formal analysis, Funding acquisition, Investigation, Methodology, Project administration, Resources, Software, Supervision, Validation, Visualization, Writing – original draft, Writing – review & editing.
