# Corrigendum: Neuroimaging in PRUNE1 syndrome: a mini-review of the literature

**DOI:** 10.3389/fneur.2024.1360347

**Published:** 2024-02-05

**Authors:** Giovanna Scorrano, Laura Battaglia, Rossana Spiaggia, Antonio Basile, Stefano Palmucci, Pietro Valerio Foti, Emanuele David, Franco Marinangeli, Ilaria Mascilini, Antonio Corsello, Francesco Comisi, Alessandro Vittori, Vincenzo Salpietro

**Affiliations:** ^1^Department of Biotechnological and Applied Clinical Sciences, University of L'Aquila, L'Aquila, Italy; ^2^Department of Medical Surgical Sciences and Advanced Technologies “GF Ingrassia”, University Hospital Policlinic “G. Rodolico-San Marco”, Catania, Italy; ^3^Department of Anesthesia, Critical Care and Pain Therapy, University of L'Aquila, L'Aquila, Italy; ^4^Department of Anesthesia and Critical Care, ARCO ROMA, Ospedale Pediatrico Bambino Gesù IRCCS, Rome, Italy; ^5^Department of Pediatrics, University of Milan, Milan, Italy; ^6^Pediatrics Department, Ospedale Microcitemico, Cagliari, Italy; ^7^Department of Neuromuscular Disorders, UCL Queen Square Institute of Neurology, London, United Kingdom

**Keywords:** *PRUNE1*, neurodevelopmental disorder, enzymatic activity, neurogenesis, delayed myelination

In the published article, there was an error in the author list, and author Antonio Corsello was erroneously excluded. The corrected author list appears below.

Giovanna Scorrano^1^, Laura Battaglia^2^, Rossana Spiaggia^2^, Antonio Basile^2^, Stefano Palmucci^2^, Pietro Valerio Foti^2^, Emanuele David^2^, Franco Marinangeli^3^, Ilaria Mascilini^4^, Antonio Corsello^5^, Francesco Comisi^6^, Alessandro Vittori^4*^ and Vincenzo Salpietro^1, 7^

^1^Department of Biotechnological and Applied Clinical Sciences, University of L'Aquila, L'Aquila, Italy

^2^Department of Medical Surgical Sciences and Advanced Technologies “GF Ingrassia”, University Hospital Policlinic “G. Rodolico-San Marco”, Catania, Italy

^3^Department of Anesthesia, Critical Care and Pain Therapy, University of L'Aquila, L'Aquila, Italy

^4^Department of Anesthesia and Critical Care, ARCO ROMA, Ospedale Pediatrico Bambino Gesù IRCCS, Rome, Italy

^5^Department of Pediatrics, University of Milan, Milan, Italy

^6^Pediatrics Department, Ospedale Microcitemico, Cagliari, Italy

^7^Department of Neuromuscular Disorders, UCL Queen Square Institute of Neurology, London, United Kingdom

In the published article, there was an error in the Author contributions statement. The contributions of Antonio Corsello were omitted due to their erroneous exclusion from the author list. The correct Author contributions statement appears below.

## Author contributions

GS: Conceptualization, Data curation, Formal analysis, Funding acquisition, Investigation, Methodology, Project administration, Resources, Software, Supervision, Validation, Visualization, Writing – original draft, Writing – review & editing. LB: Conceptualization, Validation, Visualization, Writing – original draft. RS: Data curation, Methodology, Software, Writing – original draft. AB: Formal analysis, Investigation, Software, Writing – original draft. SP: Conceptualization, Data curation, Methodology, Writing – original draft. PF: Methodology, Software, Writing – original draft. ED: Conceptualization, Investigation, Methodology, Writing – original draft. FM: Conceptualization, Data curation, Software, Writing – original draft. IM: Conceptualization, Formal analysis, Methodology, Writing – original draft. AC: Conceptualization, Data curation, Software, Writing – original draft. FC: Data curation, Investigation, Methodology, Writing – original draft. AV: Writing – original draft, Writing – review & editing. VS: Conceptualization, Data curation, Formal analysis, Funding acquisition, Investigation, Methodology, Project administration, Resources, Software, Supervision, Validation, Visualization, Writing – original draft, Writing – review & editing.

The authors apologize for this error and state that this does not change the scientific conclusions of the article in any way. The original article has been updated.

